# Structural and Functional Determinants of Rodent and Human Surfactant Protein A: A Synthesis of Binding and Computational Data

**DOI:** 10.3389/fimmu.2019.02613

**Published:** 2019-11-07

**Authors:** Armen Nalian, Todd M. Umstead, Ching-Hui Yang, Patricia Silveyra, Neal J. Thomas, Joanna Floros, Francis X. McCormack, Zissis C. Chroneos

**Affiliations:** ^1^Department of Biology, Stephen F. Austin State University, Nacogdoches, TX, United States; ^2^The Center of Biomedical Research, University of Texas Health Science Center at Tyler, Tyler, TX, United States; ^3^Department of Pediatrics, Pennsylvania State University College of Medicine and PennState Health Children's Hospital, Hershey, PA, United States; ^4^Pulmonary Immunology and Physiology Laboratory, Pennsylvania State University College of Medicine and PennState Health Children's Hospital, Hershey, PA, United States; ^5^Department of Public Health Sciences, Pennsylvania State University College of Medicine and PennState Health Children's Hospital, Hershey, PA, United States; ^6^Center of Host Defense and Inflammatory Disease Research, Pennsylvania State University College of Medicine and PennState Health Children's Hospital, Hershey, PA, United States; ^7^Department of Obstetrics and Gynecology, Pennsylvania State University College of Medicine and PennState Health Children's Hospital, Hershey, PA, United States; ^8^Division of Pulmonary, Critical Care, and Sleep Medicine, Department of Internal Medicine, University of Cincinnati College of Medicine, Cincinnati, OH, United States; ^9^Department of Microbiology and Immunology, Pennsylvania State University College of Medicine and PennState Health Children's Hospital, Hershey, PA, United States

**Keywords:** alveolar macrophages, binding, lung, surfactant protein A, receptor

## Abstract

Surfactant protein A (SP-A) provides surfactant stability, first line host defense, and lung homeostasis by binding surfactant phospholipids, pathogens, alveolar macrophages (AMs), and epithelial cells. Non-primates express one SP-A protein whereas humans express two: SP-A1 and SP-A2 with core intra- and inter-species differences in the collagen-like domain. Here, we used macrophages and solid phase binding assays to discern structural correlates of rat (r) and human (h) SP-A function. Binding assays using recombinant rSP-A expressed in insect cells showed that lack of proline hydroxylation, truncations of amino-terminal oligomerization domains, and site-directed serine (S) or alanine (A) mutagenesis of cysteine 6 (C6S), glutamate 195 (E195A), and glutamate 171 (E171A) in the carbohydrate recognition domain (CRD) all impaired SP-A binding. Replacement of arginine 197 with alanine found in hSP-A (R197A), however, restored the binding of hydroxyproline-deficient rSP-A to the SP-A receptor SP-R210 similar to native rat and human SP-A. *In silico* calculation of Ca^++^ coordination bond length and solvent accessibility surface area revealed that the “humanized” R197A substitution alters topology and solvent accessibility of the Ca^++^ coordination residues of the CRD domain. Binding assays in mouse AMs that were exposed to either endogenous SP-A or hSP-A1 (6A^2^) and hSP-A2 (1A^0^) isoforms *in vivo* revealed that mouse SP-A is a functional hybrid of hSP-A1 and hSP-A2 in regulating SP-A receptor occupancy and binding affinity. Binding assays using neonatal and adult human AMs indicates that the interaction of SP-A1 and SP-A2 with AMs is developmentally regulated. Furthermore, our data indicate that the auxiliary ion coordination loop encompassing the conserved E171 residue may comprise a conserved site of interaction with macrophages, and SP-R210 specifically, that merits further investigation to discern conserved and divergent SP-A functions between species. In summary, our findings support the notion that complex structural adaptation of SP-A regulate conserved and species specific AM functions in vertebrates.

## Introduction

Surfactant protein A (SP-A) is the most abundant lipid binding and immune-surveillance component of pulmonary surfactant. SP-A belongs to the collectin family of proteins consisting of four structural domains that include an amino-terminal tail, a collagen-like domain (CDM) with Gly-X-Y repeats, an α-helical coiled-coil neck domain with heptad repeats, and a Ca^++^-dependent carbohydrate recognition domain (CRD) (1–3). The neck and collagen-like domains of SP-A trimerize and assemble into higher order deca-octamers via inter-chain disulfide bonds in the amino-terminal tail and non-covalent interactions between trimers. Binding to extracellular ligands depends on the neck-CRD trimer and high avidity multivalent binding of the SP-A deca-octamer. Crystallographic and molecular modeling studies of the rat SP-A CRD revealed that conformational flexibility of the Ca^++^ coordination site and CRD surface loops impart versatility in the ability of SP-A to bind structurally diverse endogenous and pathogen-derived ligands (2, 4–7). The interaction of SP-A with AMs and epithelial cells is receptor-mediated (8–10). Deletion, site-directed mutagenesis, and ligand competition studies showed that the amino-terminal and collagen-like domains influence receptor occupancy and functional responses in alveolar type II epithelial cells and macrophages, and interaction of the CRD domain with surfactant phospholipids (10–16). SP-A binds dipalmitoyl phosphatidylcholine, the major surfactant phospholipid, that is required for the formation of tubular myelin from secreted lamellar bodies, contributes to the adsorption of surface active phospholipids at the air-liquid interface, and facilitates turnover of spent surfactant vesicles by alveolar type II epithelial cells through the p63 receptor (7, 17–21). In host defense, SP-A binds different pathogen ligands such as the lipid A portion of lipopolysaccharide on Gram-negative bacteria (7), surface proteins and glycolipids on gram positive bacteria (22), mycobacteria (23, 24), and fungi (25, 26). These interactions facilitate pathogen clearance through agglutination, opsonization, and direct killing (22, 26–29). SP-A enhances opsonic and non-opsonic phagocytosis (22, 30–32), and shapes pathogen-dependent polarization of inflammatory responses through the SP-R210 SP-A receptor (aka Myo18A or CD245) (22, 27, 33) in macrophages.

Unlike non-primate amniotes (34), humans express two SP-A protein isoforms, SP-A1 and SP-A2 encoded by different genes *SFTPA1* and *SFTPA2* (35), and each gene has been identified with several variants. The hSP-A1 and hSP-A2 proteins and their respective variants differ at four core amino acids in the collagen-like domain and the variants of each gene are distinguished among themselves by additional amino acid differences present in domains other than the collagen-like domain (36–38). SP-A1 and SP-A2 differentially modulate macrophage function (39–41) and in suppressing development of idiopathic interstitial pneumonia, fibrosis, and cancer (42–45). Moreover, significant differences have been observed among SP-A1 and SP-A2 variants in survival after infection and lung function (46, 47). The presence of both proteins is required for tubular myelin formation, supra-trimeric assembly of SP-A oligomers, and optimal function of surfactant (21, 48, 49) and one SP-A gene is sufficient to exert these functions in lower vertebrates (19, 50, 51). Both human and rodent SP-As have a discrete kink peptide in the middle of the CDM that confers conformational flexibility, contributes to the quaternary organization of higher order SP-A oligomers, and spatial separation of CRD domains (52–54). The kink sequence is conserved between SP-A1 and SP-A2 but different from rodent SP-A; PCPP in human SP-As and MGLP in rodents (34, 54). The kink peptide and a unique GEC collagen triplet in SP-A1 (GER in SP-A2) result in 2 cysteine residues in the CDM of SP-A1 compared to 1 in SP-A2 and none in rodent SP-A. The GEC triplet contributes to distinct oligomeric structures in SP-A1 and the two proteins distribute differently in interfacial surfactant films (48, 49, 55). Compared to SP-A2, SP-A1 improved the biophysical activity of surfactant in lowering surface tension and resistance to inhibition by serum (56). Recombinant SP-A1 lacking the ability to form oligomers, however, retains anti-inflammatory effects on macrophages (57, 58), whereas SP-A2 variants compared to SP-A1 have been shown to exhibit higher activity in bacterial phagocytosis by AMs (59) and cytokine production in a macrophage-like cell line (39, 60).To better understand SP-A function we used binding assays and molecular modeling to define molecular and functional attributes in rodent and human SP-A.

## Materials and Methods

### Animals

Wild type (SP-A^+/+^) C57BL/6J mice were purchased from JAX labs and bred locally. Transgenic SP-A^−/−^ and humanized *mSftpa*^−/−,*hSFTPA*1(6*A*2)^ and *mSftpa*^−/−,*hSFTPA*2(1*A*0)^ mice were generated as described previously (21). Sprague-Dawley rats were obtained from Harlan. All animal procedures were performed under IACUC approved protocols.

### Production and Purification of Recombinant Proteins

Recombinant rat SP-A proteins were synthesized in insect Sf9 cells via baculovirus expression of rat SP-A WT cDNA or mutant cDNAs generated by nested deletion or site directed mutagenesis as described in detail previously (25, 61). The present studies utilized rat SP-A with C6S, E171A, E195A, R197A, and D215A point mutations and deletion mutants lacking both amino terminal and collagen-like domains (ΔN1-G80), the collagen-like domain (ΔG8-G80), kink (ΔG8-G40) and proximal (ΔG40-G80) halves of the collagen-like domain. Recombinant rat SP-A proteins were isolated by affinity chromatography over mannose-Sepharose. Native rat SP-A was isolated from the bronchoalveolar lavage (BAL) of Sprague-Dawley rats 4 weeks after instillation of 40 mg/Kg of silica-induced alveolar proteinosis Rat BAL surfactant was separated by NaBr-density gradient centrifugation followed by delipidation, affinity and gel exclusion chromatography (12, 19, 25, 61–64). Native human SP-A was isolated from discarded alveolar proteinosis BAL by sequential isobutanol/β-octylglucoside extraction, repeated precipitation/solubilization in 20 mM CaCl_2_/EDTA, and extensive dialysis in 5 mM HEPES, pH 7.4 (32). Recombinant human SP-A1 (variant 6A^2^) and SP-A2 (variant 1A^0^) were expressed in CHO-K1 cells via the pEE14 expression vectors (39). SP-A1 and SP-A2 secreted in media were purified by affinity chromatography over mannose-Sepharose and gel filtration chromatography over Superose 6 (39). The purity of rat and human proteinosis SP-A was >95% and recombinant human SP-A1 and SP-A2 was >95% pure as assessed by 1 and/or 2-dimensional SDS-PAGE (39, 65), respectively. All SP-A preparations contained negligible amounts of bacterial endotoxin lipopolysaccharide below 0.1 pg/mg of protein as assessed by the Limulus Amoebocyte Lysate assay.

### Isolation of Alveolar Macrophages From Transgenic Mice and Human Lung

Mouse AMs were isolated by BAL using five sequential intratracheal instillation of 0.5 mL PBS/1 mM EDTA for a total of 2.5 mL. The BAL was centrifuged at 250 × g for 10 min to pellet the macrophages. The macrophages were washed once in PBS, counted, and then suspended in iodination blocking buffer. Human AMs were isolated from donated human lungs which were rejected for transplant *ex vivo* as described previously (66, 67). All procedures were approved by the Penn State College of Medicine Institutional Review Board. Cell purity was assessed microscopically after cytospin centrifugation and HEMA-3 differential staining (29).

### Molecular Dynamics Simulation

The starting coordinates for molecular dynamics (MD) simulations were obtained from the recombinant rat SP-A crystal structure 1R13 (4) (http://www.rcsb.org/pdb/explore.do?structureId=1r13) consisting of the neck and CRD domain with a mutation at the glycosylation site consensus asparagine 187 (N187S) and lacking the amino-terminal and collagen-like domains (ΔN1-80) using Swiss-Pdb Viewer (68). Molecular dynamics simulations were carried out as described previously (69). We tested the impact of alanine point mutations in the primary Ca^++^ coordination residues of the CRD carbohydrate-lipid-LPS (CLL) binding pocket, E195A, R197A, N214A, and D215A, and E171A in the auxiliary metal ion coordination loop on Ca^++^- coordination bond length and solvent accessibility surface area. All residues tested are conserved between rodent and human SP-A, except for R197 which is naturally substituted by an alanine in both human SP-A1 and SPA2.

### Sequence Alignment and Numbering of Rodent and Human SP-A

**Figure 1:**
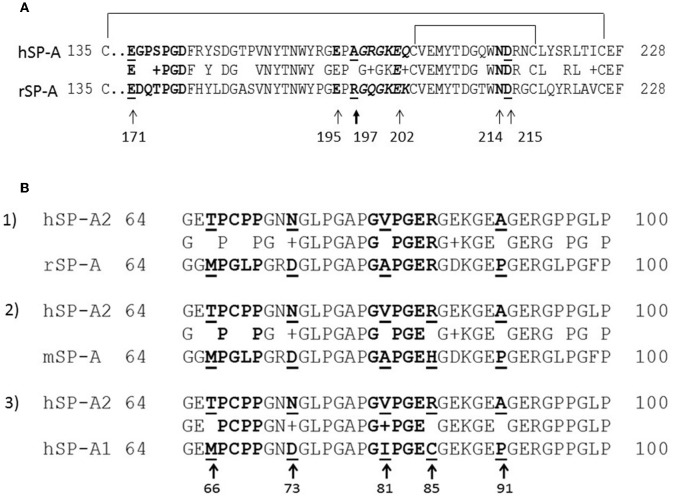
Partial sequence alignment of the CRD **(A)** and CDM **(B)** domains of rodent and human SP-A. **(A)** Alignment of the CRD domains of human SP-A2 1A^0^ variant (hSP-A2) and rat SP-A carbohydrate recognition domain. Amino acid numbering is based on the mature peptide of rat SP-A. Arrows point to Ca^++^-coordination residues. **(B)** Alignment of the collagen-like domains of human SP-A1 (6A^2^) (hSP-A1) and hSP-A2 1A^0^ variant, mouse SP-A (mSP-A), and rat SP-A (rSP-A). All alignments are made to hSP-A2 (1A^0^). The aligned sequences shown encompass the kink peptide (bolded residues 66–50) to the end of the CDM. Arrows point to core amino acids in the CDM that distinguish SP-A1 and SP-A2; the numbering is based on the precursor molecule. Residues shown in italicized red font show the sequence of embedded integrin binding motifs in the CDM. Sequence alignments were performed using ClustalW.

Sequence alignments were performed using ClustalW (70, 71) (Figure 1). The amino acid alignment and location of Ca^++^- coordination residues of rat SP-A and human SP-A2 (variant 1A^0^) are shown on Figure 1A and part of the CDM on Figure 1B. The numbering of amino acid residues on Figure 1A is based on the mature rat SP-A after cleavage of the respective signal peptide between residues C20-N21 or as stated otherwise (Uniprot Accession No: P08427) (72, 73). In humans, although the signal peptide cleavage has been shown to affect a few amino acids (74), here we based the numbering on one of the cleaved variants in C20-E21 of the SP-A2 variant 1A^0^ (Uniprot Accession No: Q8IWL1). For the CDM alignments numbering is from the start of respective signal sequences (Figure 1B) to avoid confusion with differences in amino-acid numbering for rat and human SP-A1 and SP-A2 in the literature. The numbering of all rat SP-A point and deletion mutants is based on the mature rat peptide.

### Macrophage and Solid Phase Binding Assays

Macrophage binding assays were performed using mouse or human AMs, or the RAW264.7 macrophage cell line and ^125^I-labeled SP-A as described in detail previously (10, 32). The purified His-tagged SP-A binding neck domain of SP-R210 (32, 75) was used in solid phase binding assays with SP-A. Flat bottomed 96 well ELISA plates were treated with 0.1 M Na_2_CO_3_, pH9.3, and coated with 10 μg/mL of purified nSP-R210, and blocked in 0.1% BSA. Recombinant rat SP-A, native rat, or human SP-A1 or SP-A2 were added at increasing concentration in SP-A binding buffer (HBSS not containing phenol red, 20 mM HEPES (pH 7.4), 1 mM CaCl_2_, 0.2 mM MgCl_2_, 1% bovine serum albumin) and incubated at 37°C in a humidified chamber. Plates were washed in binding buffer, incubated with HRP-conjugated anti-rat affinity purified polyclonal rabbit anti-SP-A antibody for 1 hr. at room temperature, and then washed. Bound antibody was visualized using 3, 3′, 5, 5′-tetramethylbenzidine (TMB) HRP substrate at 450 nM.

### Data Analysis

Graphical analyses of binding data were performed with GraphPad Prism software (GraphPad Software, San Diego, CA). Binding data were analyzed by non-linear regression of saturation curves fitted to a single site equilibrium binding equation using Prism software to calculate the number of SP-A binding sites/cell (B_max_) and binding affinities (K_d_). The hill equation embedded in Prizm software was used to calculate the Hill coefficient of cooperativity for human SP-A binding to murine and human alveolar macrophages.

## Results

### Spatial Interaction of Oligomerization and CRD Domains of SP-A

**Figure 2:**
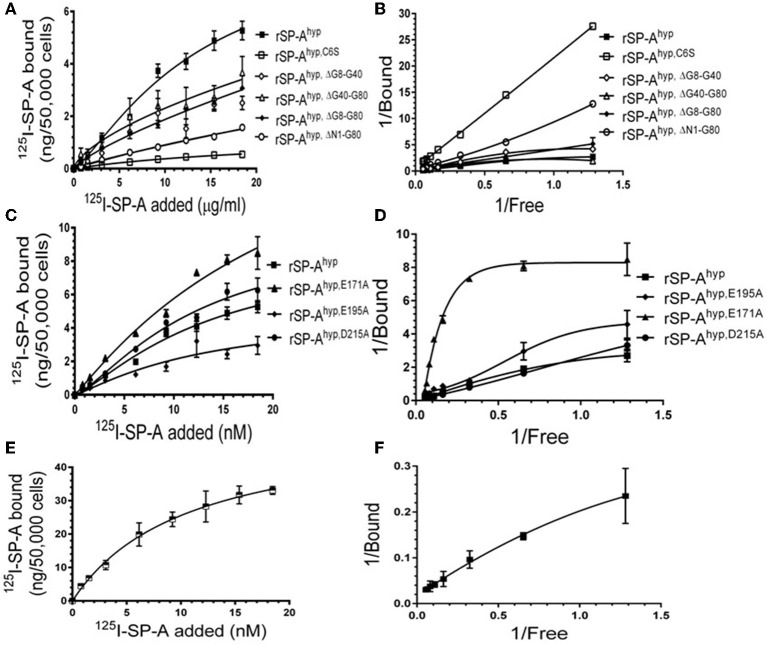
Impact of domain deletion and site directed mutagenesis on SP-A binding to macrophages. Binding assays used Raw264.7 macrophages in suspension. Cells were incubated with increasing concentrations of either radiolabeled recombinant rat SP-A proteins **(A–D)** or human SP-A **(E,F)** purified from alveolar proteinosis lavage. Binding data were fitted by non-linear regression to generate binding isotherms **(A,C,E)** or transformed into double reciprocal plots to evaluate SP-A binding behavior **(B,D,F)**. Assays were carried out at 4°C for 1.5 h. at the 50,0000 cells/assay. Bound SP-A was separated by centrifugation over a silicon oil mixture to separate bound from free SP-A. Assays were performed in triplicate and data pooled from 2 independent experiments. Data shown are Means ± SE.

**Figure 3:**
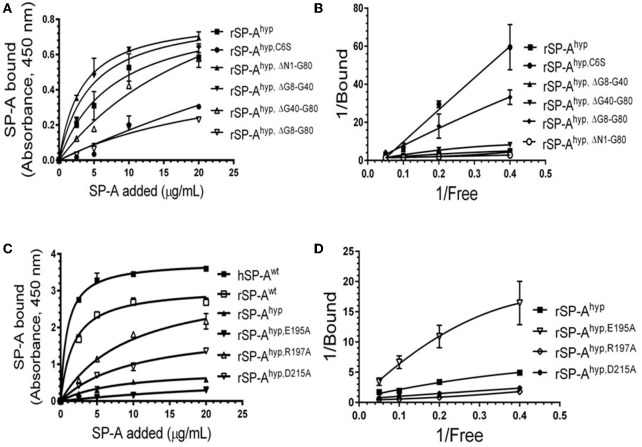
Impact of domain deletion and site-directed mutagenesis on SP-A binding to SP-R210. The recombinant SP-A binding domain of SP-R210 was coated onto microtiter well plates, and incubated with increasing concentration of indicated recombinant rat SP-A proteins **(A–D)**, or native human or rat SP-A proteins **(C,D)**. Bound protein was detected using an HRP-conjugated SP-A antibody. Binding data were fitted by non-linear regression to generate binding isotherms **(A,C)** or transformed into double reciprocal plots to evaluate SP-A binding behavior **(B,D)**. Assays were performed in duplicate or triplicate and data pooled from 2 independent experiments. Data shown are Means ± SE.

We used deletion and point mutants of insect cell expressed rat SP-A to map SP-A binding to macrophages and recombinant SP-R210 compared to native human or rat SP-A isolated from BAL. Insect cell expressed SP-A lacks proline hydroxylation of Gly-X-Pro repeats in the CDM of SP-A (rSP-A^hyp^) and also exhibits an altered pattern of glycosylation (61). Hydroxyproline imparts thermal stability that influences the degree of oligomerization of the collagen-like domain of SP-A (61). Saturation binding assays demonstrated that lack of hydroxyproline reduced SP-A binding potential to both macrophages (Figures 2A,C,E,F, Table 1) and recombinant SP-R210 (Figures 3A–D, Table 2) as demonstrated by 3–9 fold decreases in both maximal binding (Bmax) and binding affinity (Kd) compared to native SP-A.

**Table 1:** Parameters of SP-A binding to macrophages.

**Protein**	**Bmax** **(ng/50,000 cells)**	**Kd (nM)**
hSP-A	52.83 ± 0.01	6.94 ± 0.002
rSP-A^hyp^	8.94 ± 2.71	13.60 ± 6.60
rSP-A^hyp,ΔN1−G80^	7.12 ± 1.80	68.57 ± 20.59
rSP-A^hyp,ΔG8−G40^	4.68 ± 1.14	16.10 ± 6.90
rSP-A^hyp,ΔG40−G80^	3.21 ± 0.93	7.84 ± 4.04
rSP-A^hyp,ΔG8−G80^	8.58 ± 7.10	34.42 ± 39.81
rSP-A^hyp,E171A^	24.96 ± 6.00	33.56 ± 11.35
rSP-A^hyp,E195A^	5.05 ± 4.64	13.22 ± 21.16
rSP-A^hyp,D215A^	19.38 ± 5.82	36.29 ± 19.56
rSP-A^hyp,C6S^	1.01 ± 0.25	15.62 ± 6.52

**Table 2:** Parameters of SP-A binding to SP-R210.

**Protein**	**Bmax (A405)**	**Kd (nM)**
hSP-A	3.78 ± 0.08	0.90 ± 0.12
rSP-A	3.08 ± 0.11	1.76 ± 0.28
rSP-A^hyp^	0.86 ± 0.13	7.81 ± 2.60
rSP-A^hyp,ΔN1−G80^	0.82 ± 0.02	3.19 ± 0.27
rSP-A^hyp,ΔG8−G40^	0.92 ± 0.20	4.54 ± 2.60
rSP-A^hyp,ΔG40−G80^	1.32 ± 0.40	24.77 ± 11.70
rSP-A^hyp,ΔG8−G80^	0.57 ± 0.31	27.80 ± 22.75
rSP-A^hyp,E195A^	0.82 ± 0.43	36.27 ± 26.02
rSP-A^hyp,R197A^	3.31 ± 0.08	9.64 ± 2.03
rSP-A^hyp,D215A^	2.01 ± 0.18	10.10 ± 1.88
rSP-A^hyp,C6S^	0.32 ± 0.07	8.66 ± 1.77

**Figure 4:**
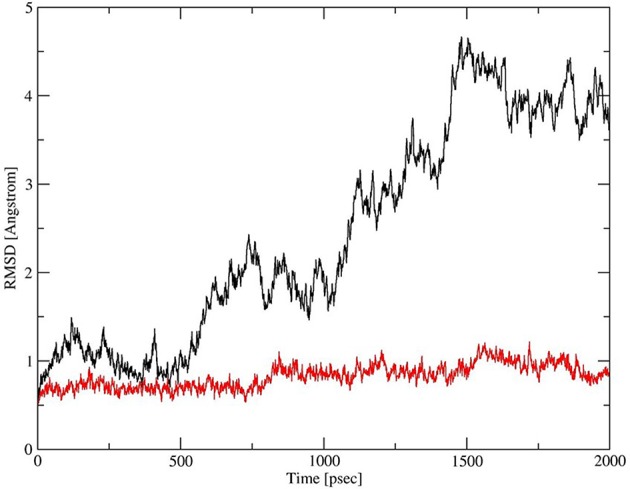
Dynamics of CRD and neck-CRD domains. Plot of root mean square deviation (RMSD) of α-Carbon atoms over time for CRD (red trace) and neck-CRD (black trace).

Deletion and site-directed mutagenesis were then used to assess the effect of the amino-terminal and CDM oligomerization domains on the binding of rSP-A^hyp^ (Figures 1A,B, 2A, Tables 1, 2). Complete CDM deletion (rSP-A^hyp,ΔG8−G80^) impaired binding to both macrophages and SP-R210 compared to rSP-A^hyp^. Point mutation of cysteine 6 to serine in the amino terminal peptide (rSP-A^hyp,C6S^) diminished Bmax with similar binding affinity to both macrophages and SP-R210 compared to rSP-A^hyp^. The partial CDM deletion rSP-A^hyp,ΔG8−G40^ and rSP-A^hyp,ΔG40−G80^ mutants had increased high affinity binding at reduced Bmax in macrophages compared to rSP-A^hyp^. The rSP-A^hyp,ΔG40−G80^ mutant, however, exhibited reduced affinity to SP-R210, whereas rSP-A^hyp,ΔN1−G80^ bound SP-R210 with increased affinity compared to rSP-A^hyp^. The Hughes-Klotz double reciprocal plots (Figures 2B,F, 3B) indicated negative cooperativity for rSP-A^hyp^, native SP-A (Figure 2F), and the partial CDM deletion mutants rSP-A^hyp,ΔG8−G40^ and rSP-A^hyp,ΔG40−G80^ compared to non-interacting binding sites for rSP-A^hyp,C6S^, rSP-A^hyp,ΔG8−G80^ and rSP-A^hyp,ΔN1−G80^, indicating that oligomerization modulates the binding behavior of SP-A. Mutants to examine the role of the neck coiled-coil domain are not available. Molecular dynamics simulations with and without the coiled-coil domain showed as the CRD alone did not equilibrate *in silico* as indicated by the increasing root mean square deviation (RMSD) of the CRD α-carbon atoms compared to the NCRD (Figure 4).

**Figure 5:**
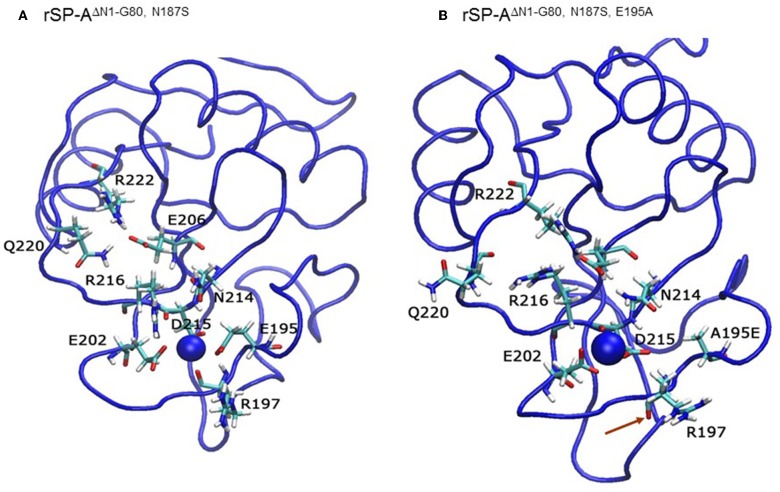
The E195A substitution alters the Ca^++^-coordination mode in the CLL binding pocket. Ribbon diagrams depicting the geometry of Ca^++^-coordination site of the native **(A)** and mutant E195A NCRD **(B)**. Red arrows point to the carbonyl oxygen in the R197 residue.

We then examined the effect of alanine (A) mutagenesis of residues that influence geometry and Ca^++^ coordination in the CRD domain (4, 5) using binding assays and MD simulations (Table 1). Compared to the crystal structure, the MD simulations indicate that the side chain carbonyl for asparagine (N) 214 and backbone carbonyl of aspartate (D) 215 occupy spaces further away from Ca^++^ in the WT CRD. The side chain hydroxyl of D215 and both oxygens in the E202 side chain are oriented closer to the Ca^++^ ion (Table 3). The invariant glutamate 171 (E171) influences Ca^++^ coordination of the E202 residue side chain, conformation of the double-loop structure surrounding the CRD carbohydrate binding pocket via a side-chain salt bridge with lysine K203, and coordination of an auxiliary ion (4, 5). The E171A substitution mutant (rSP-A^hyp,E171A^) exhibited increased binding at high concentrations to macrophages compared to rSP-A^hyp^ (Figure 2C). The double-reciprocal Hughes-Klotz plot (76) of rSP-A^hyp,E171A^ showed strongly increased negative cooperativity compared to rSP-A^hyp^ (Figure 2D), suggesting abortive interaction with macrophages at low concentration. Binding to SP-R210 was not be determined. The E202 side chain of rSP-A^hyp,E171A^ did not coordinate Ca^++^, consistent with crystallographic data (4). The average surface area solvent accessibility (SASA) of the Ca^++^ ion and Ca^++^ coordination amino acids, however, was not altered by the E171A substitution compared to WT (Tables 4, 5). In previous studies, mutation of residues E195 (rSP-A^hyp,E195A^) and D215 (rSP-A^hyp,D215A^), two invariant residues that coordinate Ca^++^, demonstrated critical roles in Ca^++^-dependent binding and aggregation of DPPC, and binding to alveolar type II epithelial cells (13). E195 coordinates Ca^++^ via its side chain oxygens. Here, the binding affinity of the rSP-A^hyp,E195A^ mutant could not be accurately determined from the binding curves (Table 1). The double-reciprocal plot of rSP-A^hyp,E195A^ binding data, however, revealed atypical sigmoidal behavior suggesting interaction with low and high affinity binding sites in macrophages (Figure 2D), whereas this mutant exhibits negative cooperativity with recombinant SP-R210 (Figure 3D). In the E195A substitution, the R197 backbone carbonyl moved 4.63 Å away from Ca^++^, and also altered the orientation of asparagine (N) 214, D215, and E202 side chains relative to the Ca^++^ ion (Table 3 and Figures 5A,B). These changes were accompanied by increased H_2_O occupancy and average SASA of the Ca^++^ coordination site residues (Tables 4, 5), suggesting that H_2_O modifies the ligand binding properties of this mutant. The rSP-A^hyp,D215A^ mutant, on the other hand, displayed increased Bmax and low affinity non-cooperative binding compared to rSP-A^hyp^ (Figures 2C, 3C and Tables 1, 2). The D215 residue contributes both its side chain and peptide bond carbonyl to Ca^++^-coordination. The *in silico* calculations showed that the D215 backbone carbonyl moves closer to Ca^++^ by 1.33 Å when the side chain is replaced by Alanine (Table 3). The D215A substitution also influenced the orientation of E195 and E202 side chains, Ca^++^ proximity of the R197 carbonyl, and increased Ca^++^-H_2_O occupancy (Tables 3–5). The arginine (R) 197 residue, which coordinates Ca^++^-H_2_O occupancy via its peptide bond carbonyl, is naturally switched with alanine in human SP-A. Interestingly, the R197A substitution increased the binding of rSP-A^hyp,R197A^ to SP-R210 similar to the level of native rat and human SP-A, neutralizing the negative impact of proline hydroxylation deficiency in the CDM (Figure 3C and Table 2). The computational analysis revealed that the R197A switch brings the hydroxyl oxygens of E195 and N214 side chains and peptide chain carbonyl oxygen of D215 closer to Ca^++^ by 1-2.5 Å while the hydroxyl oxygen of D215 and both side chain oxygens of E202 orient away from Ca^++^ (Table 3). The hydroxyl oxygen of the D215 side chain rotated the farthest, 4.15 Å away from Ca^++^ compared to WT. These adjustments in Ca^++^ coordination are accompanied by >50% decrease in hydrophilic SASA of the Ca^++^ coordination shell from 227.38 ± 23.94 to 106 ± 11.50 Å^2^ of WT and rSP-A^hyp,R197A^, respectively, suggesting a more compact structure of the “humanized” CRD (Tables 4, 5).

**Table 3:** Calcium coordination bond length in WT and mutant rat SP-A NCRD.

	**X-ray** **(ΔN1-G80)**	**WT NCRD** **(ΔN1-G80)**	**E171A**	**E195A**	**R197A**	**N214A**	**D215A**
E195:OE1	2.43	2.14 ± 0.05	2.15 ± 0.06		2.73 ± 0.71	2.17 ± 0.07	2.19 ± 0.07
E195:OE2	3.73	3.77 ± 0.2	3.63 ± 0.36		2.64 ± 0.7	3.32 ± 0.53	2.31 ± 0.13
R197:O	2.87	2.3 ± 0.09	2.27 ± 0.07	6.93 ± 0.32	2.25 ± 0.08	2.28 ± 0.09	5.82 ± 1.03
N214:ON1	2.46	4.61 ± 0.58	4.62 ± 0.39	5.12 ± 0.50	2.25 ± 0.08		4.15 ± 0.41
D215:O	2.34	3.75 ± 0.22	4.2 ± 0.28	3.63 ± 0.70	2.77 ± 0.52	4.54 ± 0.26	2.42 ± 0.26
D215:OD1	2.27	2.22 ± 0.08	2.19 ± 0.07	2.11 ± 0.05	2.13 ± 0.05	2.19 ± 0.07	
D215:OD2	4.03	2.25 ± 0.1	2.29 ± 0.13	3.95 ± 0.17	4.09 ± 0.17	2.27 ± 0.12	
E202:OE1	5.91	3.91 ± 0.2		2.22 ± 0.09	5.11 ± 1.07	4.25 ± 0.12	2.23 ± 0.12
E202:OE2	4.25	2.13 ± 0.05		2.23 ± 0.09	6.28 ± 0.51	2.14 ± 0.06	2.35 ± 0.42

**Table 4:** Effect of CRD mutation on binding pocket water occupancy.

**Protein**	**% of time within 3Å**				**SASA of Ca^**++**^**
	**1**	**2**	**3**	**4**	
WT	100%	100%			4.92 ± 1.89
E171A	100%	98%			5.07 ± 2.32
E195A	100%	73%	73%	67%	27.74 ± 5.03
R197A	100%	100%			7.02 ± 1.62
N214A	10%	98%			6.12 ± 2.38
D215A	100%	100%	87%		11.10 ± 2.57
X-Ray	+	+			

**Table 5:** Effect of mutation on SASA of Ca^++^ coordination residues.

**Protein**	
WT	227.38 ± 23.94
E171A	192.57 ± 20.65
E195A	220.28 ± 16.24
R197A	106.33 ± 11.50
N214A	194.83 ± 15.71
D215A	231.82 ± 28.77
X-Ray	265.76

### Cross-Species Paracrine Effects of Mouse and Human SP-A on AMs

**Figure 6:**
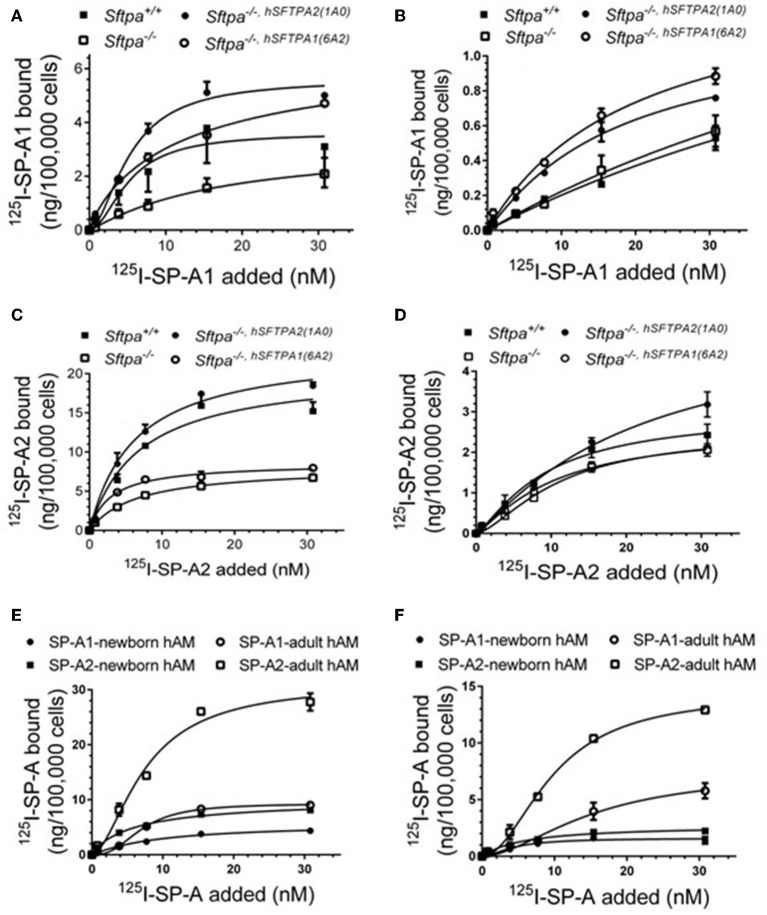
Murine and human SP-A variants are paracrine regulators of SP-A binding to alveolar macrophages in postnatal lung. Binding assays were carried out with alveolar macrophages obtained from either murine lung **(A–D)** or human lung **(E,F)** using radiolabeled human SP-A1 or SP-A2 in the presence **(A,C,E)** or absence of calcium **(B,D,F)**. Alveolar macrophages from murine lungs **(A–D)** were obtained from WT (SP-A^+/+^), SP-A-deficient (SP-A^−/−^), or humanized mice expressing either SP-A1 variant 1A^0^ (SP-A^−/−,hSFTPA2(1A0)^ ), or SP-A2 variant 6A^2^ (SP-A^−/−,hSFTPA2(6A2)^ ) in the absence of endogenous murine SP-A (*Sftpa*). Human alveolar macrophages were obtained from 6 month **(E)** and 20 years old **(F)** rejected transplant lungs. Assays were carried out at 4°C for 1.5 h at 50,0000 cells/assay. Bound SP-A was separated by centrifugation over a silicon oil mixture. Assays were performed in duplicate and data pooled from 2 independent experiments. Alveolar macrophages from mouse lungs were pooled from 10 mice per genotype to obtain sufficient number of cells per experiment. Binding isotherms were generated using Prizm software by non-linear regression analysis using the Hill equation. Data shown are Means ± SE.

We took advantage of previously developed “humanized” SP-A transgenic mice to determine the impact of SP-A isoforms on AM binding phenotype. The hSP-A1 and hSP-A2 isoforms were expressed in the lungs of SP-A^−/−^ mice. Binding assays were carried out in AMs from WT (SP-A^+/+^), SP-A-deficient (SP-A^−/−^), or SP-A^−/−^ mice carrying either expression of human SP-A genes *SFTPA1 (6A*^2^*) or SFTPA2 (1A*^0^*)* via the alveolar epithelial *Sftpc* promoter (21). The coding sequences of 6A^2^ and 1A^0^ hSP-A1 and hSP-A2 variants, respectively, differ in four core amino acids in their collagen-like domain plus amino acids 19 and 91 (77), based on the numbering of the precursor molecule. The binding assays in Figures 6A–D and Table 6 show that *in vivo* expression of hSP-A2 resulted in ligand-induced upregulation of hSPA2 binding to AMs compared to SP-A^−/−^ and hSP-A1 exposed AMs as demonstrated by the 2-2.5 increase in Bmax (Table 6). hSP-A2 also improved the binding potential of hSP-A1 by increasing binding affinity. hSPA1 did not bind SP-A^−/−^ AMs and its expression alone induced only low affinity binding of hSPA1 compared to high affinity binding for hSP-A2, although at lower Bmax compared to the hSPA2 expression. High affinity binding of hSP-A1 was observed only in AMs from hSP-A2 or WT mice. The endogenous SP-A, however, had a hybrid effect enhancing both binding affinity and Bmax with positive cooperativity. Compared to one another, the Bmax of hSP-A1 binding was 2–3-fold lower than that of hSP-A2 binding to murine AMs, indicating that the CDM is responsible for differences in receptor binding capacity of hSP-A isoforms with identical CRD.

**Table 6:** Parameters of human SP-A binding to murine alveolar macrophages.

	**SP-A1**			**SP-A2**		
**Genotype**	**Bmax**	**Kd (nM)**	**h**	**Bmax**	**Kd (nM)**	**h**
SP-A^+/+^	3.60 ± 1.32	5.14 ± 3.13	1.88 ± 2.3	16.57 ± 1.30	5.00 ± 0.70	1.88 ± 0.49
SP-A^−/−^	No binding	–	–	8.43 ± 1.40	7.04 ± 3.00	0.93 ± 0.20
SP-A*^−/−,*Sftpa*2(1*A*0)^*	5.60 ± 0.54	5.29 ± 0.17	1.76 ± 0.47	20.37 ± 1.31	5.00 ± 0.67	1.36 ± 0.22
SP-A*^−/−,*Sftpa*1(6*A*2)^*	6.84 ± 1.45	12.96 ± 7.01	0.89 ± 0.17	8.10 ± 0.45	2.80 ± 0.47	1.25 ± 0.21
	**SP-A1+EDTA**			**SP-A2+EDTA**		
SP-A^+/+^	No binding	–	–	3.00 ± 0.42	9.20 ± 2.52	1.29 ± 0.27
SP-A^−/−^	No binding	–	–	2.50 ± 0.28	10.82 ± 2.04	1.53 ± 0.26
SP-A*^−/−,*Sftpa*2(1*A*0)^*	1.19 ± 0.33	17.63 ± 9.85	1.07 ± 0.23	5.32 ± 1.60	21.40 ± 11.95	1.14 ± 0.23
SP-A*^−/−,*Sftpa*1(6*A*2)^*	1.66 ± 0.62	26.20 ± 21.13	0.92 ± 0.19	2.60 ± 0.28	10.26 ± 2.16	1.24 ± 0.17

To address differences in receptor binding properties between mouse and human AMs, we performed binding assays with human AMs obtained from donated non-transplanted lung from a 6 month old newborn and a 20 year old adult individual. Figure 6E and Table 7 demonstrate that the Bmax for hSP-A2 was 3-fold higher in adult AMs than newborn AMs compared to a 1.6-fold increase for hSP-A1. The binding affinities were similar. Non-linear regression analysis of saturation data (Figure 6E, Table 7) revealed a change from non-cooperative to cooperative binding in newborn vs. adult AMs, respectively. The binding assays in mouse AMs exposed to endogenous or human SP-As *in vivo* indicate that hSP-A2 and endogenous mouse SP-A shaped the positive cooperativity binding phenotype of AMs (Figures 6A,B, and Table 6), whereas hSP-A1 did not. Additional assays in the presence of EDTA revealed that 30–50% of Bmax for hSP-A1 and hSP-A2 represent Ca^++^-independent binding sites (Figure 6F). The cooperative binding behavior in the absence of Ca^++^ was not altered. The binding affinities for both hSP-A isoforms were higher in newborn AMs compared to adult AMs, suggesting developmental regulation of SP-A binding in postnatal life.

**Table 7:** Parameters of human SP-A binding to human alveolar macrophages.

**AM source**	**SP-A1**			**SP-A2**		
	**Bmax**	**Kd (nM)**	**h**	**Bmax**	**Kd (nM)**	**h**
Newborn AM	5.74 ± 0.75	9.55 ± 2.84	1.06 ± 0.17	10.23 ± 1.30	6.42 ± 0.70	0.91 ± 0.49
Adult AM	9.30 ± 0.34	7.02 ± 0.40	2.61 ± 0.34	31.12 ± 2.62	7.46 ± 1.10	1.73 ± 0.34
	**SP-A1+EDTA**			**SP-A2+EDTA**		
Newborn AM	1.60 ± 0.24	4.43 ± 1.25	1.72 ± 0.90	2.74 ± 0.27	5.94 ± 1.42	1.02 ± 0.16
Adult AM	7.49 ± 2.22	15.23 ± 6.46	1.78 ± 0.65	14.36 ± 0.71	9.80 ± 0.73	1.97 ± 0.20

## Discussion

The present studies refine our understanding of structural features that modulate rodent and human SP-A binding properties to macrophages. To our knowledge, the present study is the first direct comparison of rodent and human SP-A variants. Nested deletion mutagenesis, point mutations, and *in silico* simulations of rat SP-A indicated that high affinity binding to macrophages or SP-R210 depends on inter-domain interactions and binding sites in the CDM and CRD domains. Previous studies reported that the CDM of rat and human SP-A mediates binding to macrophages (10, 14). Here, deletion of the entire CDM abolished high affinity binding to both macrophages and SP-R210, whereas the rSP-A^hyp,ΔN1−80^ mutant lacking both domains displayed low affinity binding relative to SP-A^hyp^, suggesting that the amino-terminal peptide plays a conformational role in modulating binding distally. Partial CDM deletion, however, indicated that the amino-terminal half of the CDM that follows the kink peptide binds macrophages and SP-R210. On the other hand, site directed mutagenesis in the CRD domain showed that the E171A, D215A, and E195A point mutants altered binding behavior. All three mutations altered the conformation of Ca^++^ coordination and, furthermore, D215A and E195A increased accessibility to solvent. The E171 residue stabilizes the conformation of the CRD surface loops, while D215 and E195 are critical for binding to carbohydrates, lipids, and LPS (4–7). The present findings support the notion that the SP-R210 binding site on the CRD domain lies outside the CLL binding pocket.

Species adaptation in the CDM domain produced two alternate SP-A isoforms, SP-A1 and SP-A2, and changes in the CRD produced four variants, two for SP-A1 and two for SP-A2. These demarcate differences in ligand-induced modulation of receptor occupancy on AMs *in vivo*. In the present study we studied two of the CRD variants, one for SP-A1 (6A^2^) and one for SP-A2 (1A^0^). The amino-terminal half of the CDM of rat SP-A (Figure 1B) contains contiguous ^85^RGDKGE^90^ integrin binding motifs that are partially conserved in mouse SP-A with a corresponding ^85^HGDKGE^90^ but diverged in human SP-A with ^85^CGEKGE^90^ in SP-A1 and ^85^RGEKGE^90^ in SP-A2 (Figure 1B). Although RGD, HGD, and KGE motifs are well-known for binding to integrins (78, 79), other studies have reported interactions with the C2 membrane binding domain of phospholipases and other proteins (80–82). The interaction of this motif with SP-R210 remains to be determined. Evolutionary adaptation in the CRD domain of both isoforms introduced a natural R197A substitution between rodent and human SP-A2 (1A^0^) (Figure 1A). This substitution appears to isolate the impact of post-translational hydroxylation of proline on receptor occupancy by altering the interaction of the CRD domain with solvent and topology of Ca^++^ coordination residues in the CLL. These CDM and CRD adaptations may compensate for differences in dietary requirements for ascorbic acid and proline hydroxylation between rodents and humans (83). The present findings, however, indicate that single amino acid changes can profoundly affect CRD conformation, domain-domain interaction, and binding to macrophage receptors via CDM and CRD domains. In this regard, the CRD domain of human SP-A1 and SP-A2 is polymorphic resulting in diverse allelic combinations of SP-A1 and SP-A2 variants. Both coding and non-coding polymorphisms have been associated with increased or decreased risk to lung disease in numerous studies (77, 84). Understanding the structure-function relationship on human SP-A isoforms will require better understanding of copy number variation and biochemical characterization of polymorphic chain composition of SP-A1 and SP-A2 isoforms.

SP-A plays versatile roles in host defense through concomitant interactions with pathogens, surfactant lipids, and immune cells (1). SP-A controls the direction of macrophage activation, suppressing or inducing macrophage inflammatory responses as well as polarization toward either M1 or M2 types of differentiation and intrinsic regulation of innate immune receptor expression and function through the coordination of two SP-R210 isoforms (14, 27, 29, 33, 85, 86). Given present evidence that the SP-A CLL interacts with pattern recognition receptors (87), a distinct receptor binding site could be used to facilitate transfer of diverse SP-A cargo to innate immune receptors on macrophages. The SP-A structural determinants that differentially modulate receptor occupancy and signaling in AMs in the face of diverse immune challenges in the local lung microenvironment are subject to extensive investigation at the molecular level.

## Conclusions

Human SP-A isolated from alveolar proteinosis fluid has been utilized extensively to discern function utilizing rodent alveolar macrophages without knowledge of the donor SP-A genotype. These studies generated invaluable information but also produced controversial as well as disparate results on SP-A immune functions and identity of SP-A receptors. The present findings in mouse AMs exposed to human SP-A isoforms not only support cross-species conservation and validity of *ex vivo* studies but also indicate genetic origin of SP-A preparations as an important variable in understanding versatility of SP-A function in humans. In this regard, the presence of SP-A1 may attenuate receptor occupancy, which likely regulates functional interactions with AMs at physiologically high levels of SP-A at homeostasis. Furthermore, although the present studies are limited by the small numbers of samples, the findings in newborn and adult AMs suggest increased receptor complexity that is developmentally regulated in humans.

Finally, our simulation and binding data indicate that the auxiliary ion coordination loop encompassing the conserved E171 residue may comprise a conserved site of interaction with macrophages, and SP-R210 specifically, that merits further investigation to discern conserved and divergent SP-A functions between species. In this context, we recognize that there are limitations inherent to MD simulations that impact the behavior of ions. MD simulations, however, have the potential to unveil how structural differences alter dynamic properties that are crucial for understanding the interplay of structure and function as well as providing guidelines for experiments. The purpose of the simulations, however, was not to reproduce the exact theoretical or experimental ion interactions but instead, to carry out a comparative study of wild type and mutated structures and specifically investigate the distances and their stability. This comparative *in silico* analysis of the R197A mutant in combination with our binding results showing that this species adapted residue modulates binding to the receptor opens exciting questions on future structure-function studies to understand the basis of molecular adaptation of the human SP-A molecules.

## Data Availability Statement

The raw datasets supporting the conclusions of this manuscript will be made available by the authors upon request.

## Ethics Statement

The animal study was reviewed and approved by Institutional Animal Care and Use Committee, Pennsylvania State University College of Medicine. All procedures with isolation of human alveolar macrophages were approved by the Penn State College of Medicine Institutional Review Board.

## Author Contributions

AN acquired and generated figures and tables for all the molecular dynamics simulation data. TU performed all binding assays with hSP-A1 and hSP-A2, isolated macrophages from mouse and lung BAL, and purified human SP-A proteins. C-HY performed all binding assays with recombinant rat SP-A, purified recombinant SP-R210, and cultured cells. PS and NT coordinated the procurement and logistics of donated human lung and contributed to the isolation of human alveolar macrophages. JF provided humanized SP-A1 and SP-A2 mice for the isolation of alveolar macrophages, and edited the manuscript critically. FM produced all recombinant rat SP-A proteins. ZC conceptualized, designed, led the study, and wrote the manuscript.

### Conflict of Interest

The authors declare that the research was conducted in the absence of any commercial or financial relationships that could be construed as a potential conflict of interest.

## Abbreviations

AMs: alveolar macrophages
BAL: bronchoalveolar lavage
CDM: collagen-like domain
CLL: carbohydrate, lipids, LPS binding site
CRD: carbohydrate recognition domain
GEC: Glycine Glutamate Cysteine
GER: Glycine Glutamate Arginine
MDLG: Methionine Aspartate Leucine Glycine
NCRD: coiled-coil neck and CRD domain
PCPP: Proline Cysteine Proline Proline
SP-A: surfactant protein A
rSP-A: native rat SP-A
hSP-A: native human SP-A
hSP-A1 6A2: human SP-A isoform 1 variant 6A2
hSP-A2 1A0: human SP-A isoform 2 variant 1A0
rSP-Ahyp: hydroxyproline-deficient recombinant rat SP-A
SP-R210: SP-A receptor 210
Myo18A: Myosin 18A.
